# Incidence of ventral hernia surgery after laparoscopic bariatric surgery in Sweden: a registry-based study 2009–2019

**DOI:** 10.1007/s10029-025-03547-w

**Published:** 2025-12-20

**Authors:** Sandra Ahlqvist, Jakob Walldén, Johan Blixt Dackhammar, Pär Nordin, Charlotta Wadsten, Johan Ottosson, Yücel Cengiz

**Affiliations:** 1 Department of Diagnostics and Intervention (Sundsvall Hospital), Umeå University, Umeå, Sweden; 2 Department of Clinical Sciences, Danderyd Hospital, Karolinska Institutet, Stockholm, Sweden; 3 Sundsvall Hospital, Sundsvall, Sweden; 4 Department of Diagnostics and Intervention (Östersund Hospital), Umeå University, Umeå, Sweden; 5 Department of Surgery, Faculty of Medicine and Health, Örebro University, Örebro, Sweden

**Keywords:** Bariatric surgery, Complications, Incisional hernia, Laparoscopy, Port site hernia, Trocar site hernia

## Abstract

**Purpose:**

The incidence of trocar site hernia (TSH) after bariatric surgery is unclear. This study aims to describe the cumulative incidence of ventral hernia surgery after laparoscopic bariatric surgery in total and by laparoscopic method (LRYGB; Roux-en-Y Gastric Bypass and LSG; Sleeve Gastrectomy).

**Methods:**

This was a register based observational study on patients subjected to laparoscopic bariatric surgery (LRYGB or LSG) in Sweden 2009–2019. The Scandinavian Obesity Surgery Registry (SOReg) was linked to the Swedish National Patient Register (NPR) to obtain instances of ventral hernia surgery. Nearby codes were used as proxies for TSH surgery, since a specific procedure code for TSH surgery is lacking.

**Results:**

In 64 124 patients, mean follow-up was 67 ± 36 months, LRYGB (*n =* 52 020) 74 ± 34 months and LSG (*n =* 12 104) 34 ± 22 months. Mean time between bariatric- and ventral hernia surgery was 36 ± 28 months (range 0–129). The five-year cumulative incidence of surgery for ventral hernia was 2.9% (CI 2.8–3.1). The probability of having hernia surgery was significantly higher for LRYGB compared to LSG (Breslow test, *p <* 0.001), still significant with differences in follow-up time accounted for (*p <* 0.001).

**Conclusion:**

The incidence of surgery for ventral hernia after laparoscopic bariatric surgery is not negligible in this material covering over a decade of bariatric procedures. Ventral hernia surgery was more common after gastric bypass than after sleeve gastrectomy.

**Supplementary Information:**

The online version contains supplementary material available at 10.1007/s10029-025-03547-w.

## Introduction

Bariatric surgery is the most effective treatment for morbid obesity and is mostly performed laparoscopically. A complication to abdominal surgery is incisional hernia, after laparoscopy referred to as trocar site hernia (TSH). Obesity is a well-established risk factor for the development and the recurrence of ventral abdominal wall incisional hernias. Pathophysiological mechanisms that have been proposed to explain this association includes chronically increases intra-abdominal pressure, impaired wound healing and a higher incidence of postoperative complications among obese individuals [1, 2]. Trocar site hernia can be asymptomatic but has a potential to present with acute symptoms such as bowel incarceration and strangulation [3, 4]. The incidence of TSH is unclear, and varies between a few percent and up to 40%, with different study designs and follow-up times [5–9]. Imaging such as computed tomography and ultrasound tend to diagnose more TSH than are clinically detectable [10, 11]. In a systematic review of midline incisional hernias, approximately half of the hernias caused symptoms, and about one third underwent repair [12]. The proportion of symptomatic trocar site hernias remains unknown. While the use of laparoscopic surgery has dramatically increased over the last three decades, the number of TSH repairs has not been reported to match that increase. Still, with the large annual number of laparoscopic procedures for bariatric surgery as well as other laparoscopic abdominal surgeries worldwide, even a few percent symptomatic TSHs represent a significant burden in terms of patient suffering and healthcare burden.

Also the timing of incisional hernia occurrence remains unclear; however, evidence from both open and laparoscopic surgery suggests that longer follow-ups are associated with increased detection rates of incisional hernias [13, 14]. It has been suggested that early studies may underestimate the long-term incidence, and that the incisional hernias that develop later are generally less symptomatic [13, 15].

Sleeve gastrectomy is the leading bariatric technique worldwide [16]*.* Roux-en-Y Gastric Bypass has been the most popular procedure in Sweden for many years, but LSG has increased significantly from 2012 [17]. In LSG, specimen retrieval may necessitate enlargement of one trocar site, leading to a larger fascial defect than in LRYGB where the abdominal wall is left with only the original trocar incisions. The evidence is limited in the literature but suggests that LSG results in little to no difference in reoperation for incisional hernia repairs, compared with LRYGB [18]. This needs to be clarified.

The primary aim of the study was to study the incidence of ventral hernias subjected to surgical repair after laparoscopic bariatric surgery, using Sweden’s national patient registry and the Scandinavian Obesity Surgery Registry.

The secondary aim was to present a descriptive comparison of ventral hernia repair incidence between the most common methods for bariatric surgery (LRYGB and LSG).

## Materials and methods

This was a register based observational study of patients subjected to laparoscopic bariatric surgery (gastric bypass or sleeve gastrectomy) in Sweden 2009–2019 and subsequently to surgery for ventral hernia/hernia repair. The cohort was obtained from the Scandinavian Obesity Surgery Registry (SOReg) and linked to the Swedish National Patient Register (NPR) to collect KVÅ (Classification of Health Care Measures)- and ICD-10-SE- (current Swedish version of International Statistical Classification of Diseases and Related Health Problems) codes for ventral hernia surgery and/or diagnosis.

The study was approved by the Swedish Ethical Review Authority (File no. 2021–05589-02).

### Study population and study period

All patients subjected to primary laparoscopic gastric bypass or sleeve gastrectomy in Sweden between 1 January 2009 to 31 December 2019 were eligible for inclusion, regardless of age. All patients with surgeries converted to open surgery were excluded in the data retrieval from SOReg. Patients with a procedural code for ventral hernia repair (**Supplementary Table A**) within the study period but dated prior to their bariatric procedure were excluded.

Starting point for follow-up was the date for bariatric surgery. Event, date of death or 31 December 2019, whatever came first, was regarded as the end of follow-up. Emigration was not collected.

Procedures coded on the same day as the bariatric surgery were retained in the dataset but excluded from analysis. These cases were reported separately, as the data did not permit distinction between hernia repair performed during the bariatric procedure and repair performed later the same day.

Apart from the length of follow-up time, the specific inclusion period 2009–2019 was chosen to include the period in which the most laparoscopic bariatric procedures were performed in Sweden and extended to when the number of sleeve gastrectomies increased compared to when the registration in SOReg began in 2007. Because of the difference in number of procedures between the methods, a subgroup analysis was made for 2014–2019, when more than a third of the bariatric procedures were sleeve gastrectomies.

### Data sources

#### Scandinavian Obesity Surgery Registry (SOReg)

SOReg includes data from bariatric surgery in Sweden in terms of methods, surgical outcomes, complications and baseline data including relevant comorbidities and previous surgical procedures (cholecystectomy, anti-reflux surgery, obesity surgery) [19]. Date of death is included in SOReg, which the register obtains from the Population Register (Swedish: folkbokföringsregistret), administered by the Swedish Tax Agency (Skatteverket). Since 1 January 2013, all units who perform bariatric surgery in Sweden report their procedures and, for the study period, the overall acquisition rate for SOReg was 97%. The relevant variables are presented in Table 1.

**Table 1:** Patient characteristics

	Total	LRYGB	LSG	
	***n =* 64 124**	***n =* 52 020 (81.1%)**	***n =* 12 104 (18.9%)**	
**BMI at bariatric surgery (mean, SD)**	41.6 (5.5)	42.1 (5.3)	39.6 (5.8)	*p <* 0.001
**Sex, n (%)**				
**men, n (%)**	14 875/64 124 (23.2)	12 548/52 020 (24.1)	2327/12 104 (19.2)	*p <* 0.001
**women, n (%)**	49 249/64 124 (76.8)	39 472/52 020 (75.9)	9777/12 104 (80.8)	*p <* 0.001
**Age at bariatric surgery, years** **(mean, SD)**	41 (11.0)	41 (11.0)	41 (11.0)	*p <* 0.096
**OSA, n (%)**	6237/64 123 (9.6)	5356/52 019 (10.3)	881/12 104 (7.3)	*p <* 0.001
**Arterial hypertension, n (%)**	15 797/64 123 (24.6)	13 372/52 019 (25.7)	2425/12 104 (20.0)	*p <* 0.001
**Diabetes mellitus,** **n (%)**	8426/64 123 (13.1)	7350/52 019 (14.1)	1076/12 104 (8.9)	*p <* 0.001
**Actively smoking,** **n (%)**	6212/53 109 (11.7)	5216/42 005 (12.4)	993/11 104 (9.0)	*p <* 0.001

LRYGB; Laparoscopic Roux-en-Y Gastric bypass, LSG; Laparoscopic Sleeve Gastrectomy, OSA; Obstructive sleep apnea

### National Patient Register (NPR)

The National Patient Register, administered by the National Board of Health and Welfare, covers all types of nationwide inpatient admissions and outpatient encounters with physicians in specialized care, but not primary care. The NPR includes all discharge diagnosis according to the current Swedish version of The International Classification of Diseases, Tenth Revision (ICD-10-SE) and surgical intervention codes according to the Classification of Health Care Procedures (KVÅ; Classification of Health Care Measures) as reporting is mandatory for all publicly funded health care providers. The National Inpatient Register (IPR; Swedish: slutenvårdsregistret) has a coverage of almost 100% of hospital discharges; whereas the coverage of hospital-based outpatient care (Swedish; öppenvårdsregistret) is considerably lower (about 80%) [20].

A specific ICD code for trocar site hernia or a KVÅ-code for trocar site hernia repair did not exist during the timeframe of this study, and as a proxy, several codes associated to ventral hernia and surgery for ventral hernia were selected. Inguinal hernias were excluded. Parastomal hernias, although they are incisional hernias, were excluded due to their special properties. The merged dataset contained all procedure- and diagnosis codes of interest registered for each patient in the study, but only the first registered date for ICD and KVÅ, respectively, was chosen for analysis. All hernia surgeries presented were registered after the date of the obesity operation.

Procedure- and diagnosis codes requested from NPR (Supplementary Table A and Table B) are listed in the appendix.

## Statistics

**Fig. 1:**
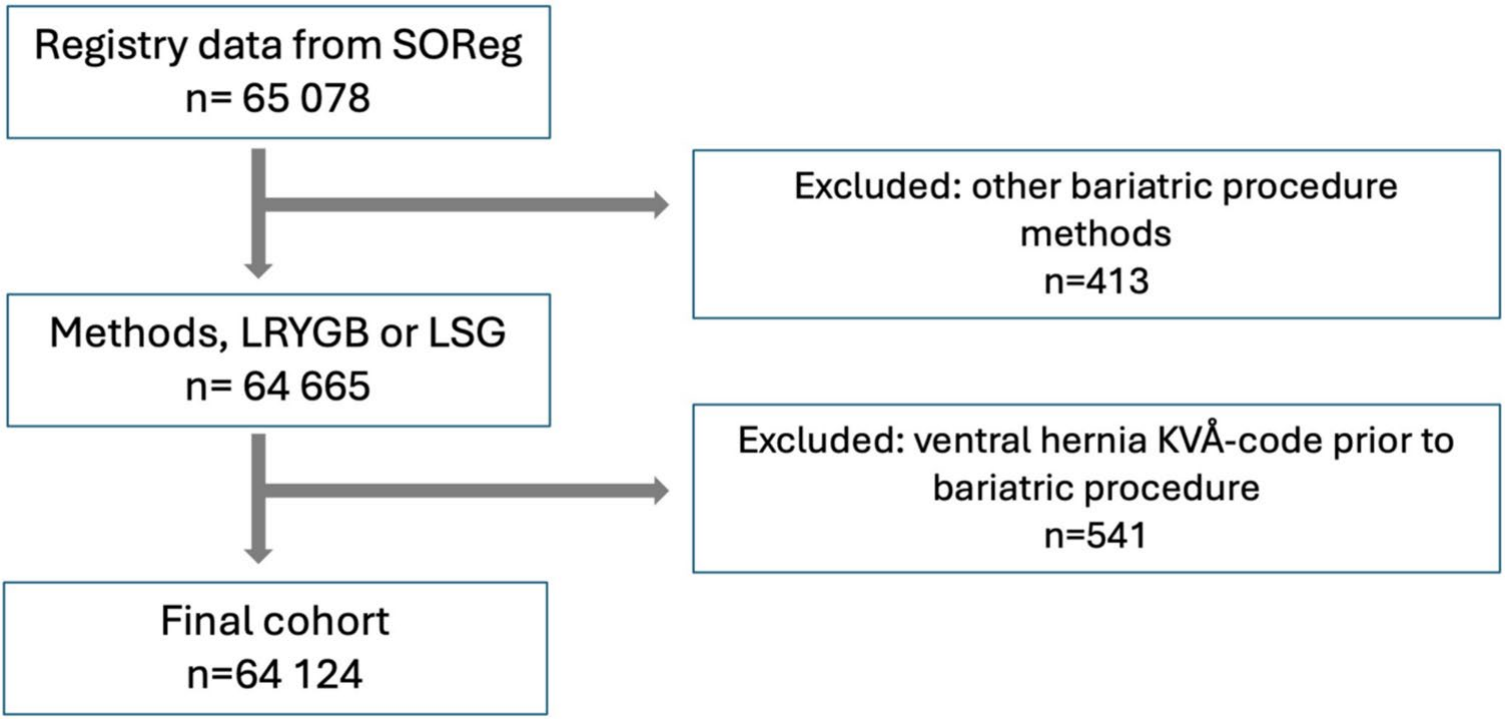
Flow chart of patient inclusion and exclusion. LRYGB; Laparoscopic Roux-en-Y Gastric bypass, LSG; Laparoscopic Sleeve Gastrectomy

Descriptive data are presented with numbers (percentages), mean (standard deviations, SD) and median (interquartile range; IQR), as appropriate. The Chi-2 test was used to compare proportions for categorical variables. The t-test was applied to compare continuous variables between groups. Our primary endpoint was time to ventral hernia repair, which occurred at identifiable exact dates and enabled a time-to-event analysis. The cumulative rate of surgery for ventral hernia was calculated using Kaplan Meier (KM), with date for bariatric surgery as starting point and censoring for death. The proportional hazards assumption was violated according to the Kaplan–Meier curves using visual inspection of the log–log plots, and the Breslow test was used. Interaction terms between method and follow-up (both linear and log-transformed) were included to model time-dependent effects. Statistical significance was defined as *p <* 0.05. Statistical analyses were performed with SPSS (IBM Corp. Released 2023. IBM SPSS Statistics for Macintosh, Version 29. Armonk, NY: IBM Corp) and RStudio Team. (RStudio: Integrated Development Environment for R. Boston: Posit Software, PBC; 2024. Available from: https://posit.co/) for Kaplan Meier (Fig. 1).

## Results

### Participants

**Fig. 2:**
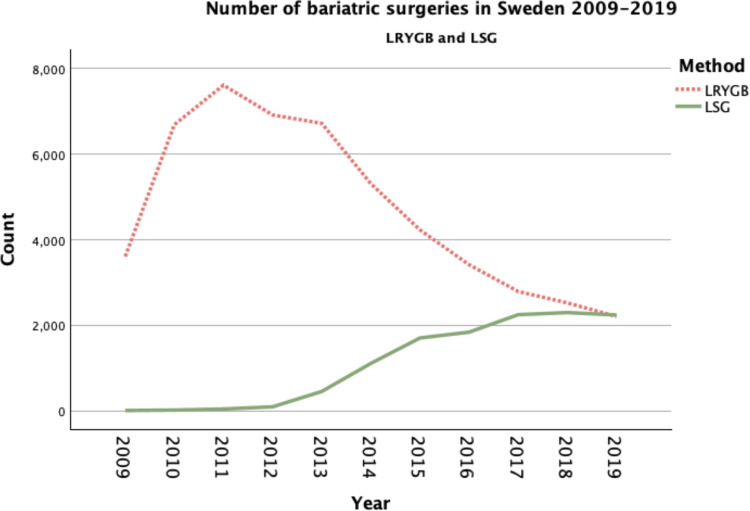
Number of bariatric surgeries in Sweden 2009–2019. LRYGB; Laparoscopic Roux-en-Y Gastric bypass, LSG; Laparoscopic Sleeve Gastrectomy

In total, 64 124 patients were included. 52 020 (81%) had undergone gastric bypass, and 12 104 (19%) sleeve gastrectomy (Table 1). The annual numbers of bariatric surgeries in Sweden 2009–2019 are shown in Fig. 2.

### Follow-up time

Mean follow-up was 67 ± 36 months in total, for LRYGB 74 ± 34 months and for LSG 34 ± 22 months. In the sub analysis for the period 2014–2019, mean follow-up was 38 ± 21 months in total, for LRYGB 42 ± 21 months and for LSG 32 ± 19 months. In total, 59 191/64 124 (92.3%) had a follow-up at least 1 year.

### Outcomes

#### Incidence of ventral hernia after laparoscopic bariatric surgery

**Fig. 3:**
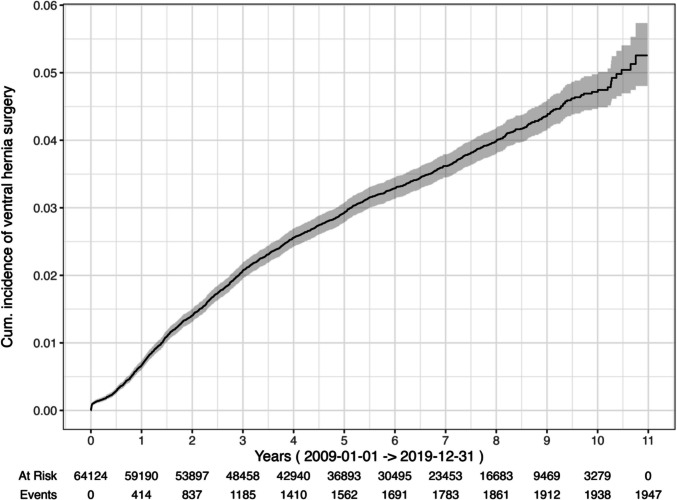
Cumulative incidence of ventral hernia surgery, total cohort, 2009–2019. LRYGB; Laparoscopic Roux-en-Y Gastric bypass, LSG; Laparoscopic Sleeve Gastrectomy

**Fig. 4:**
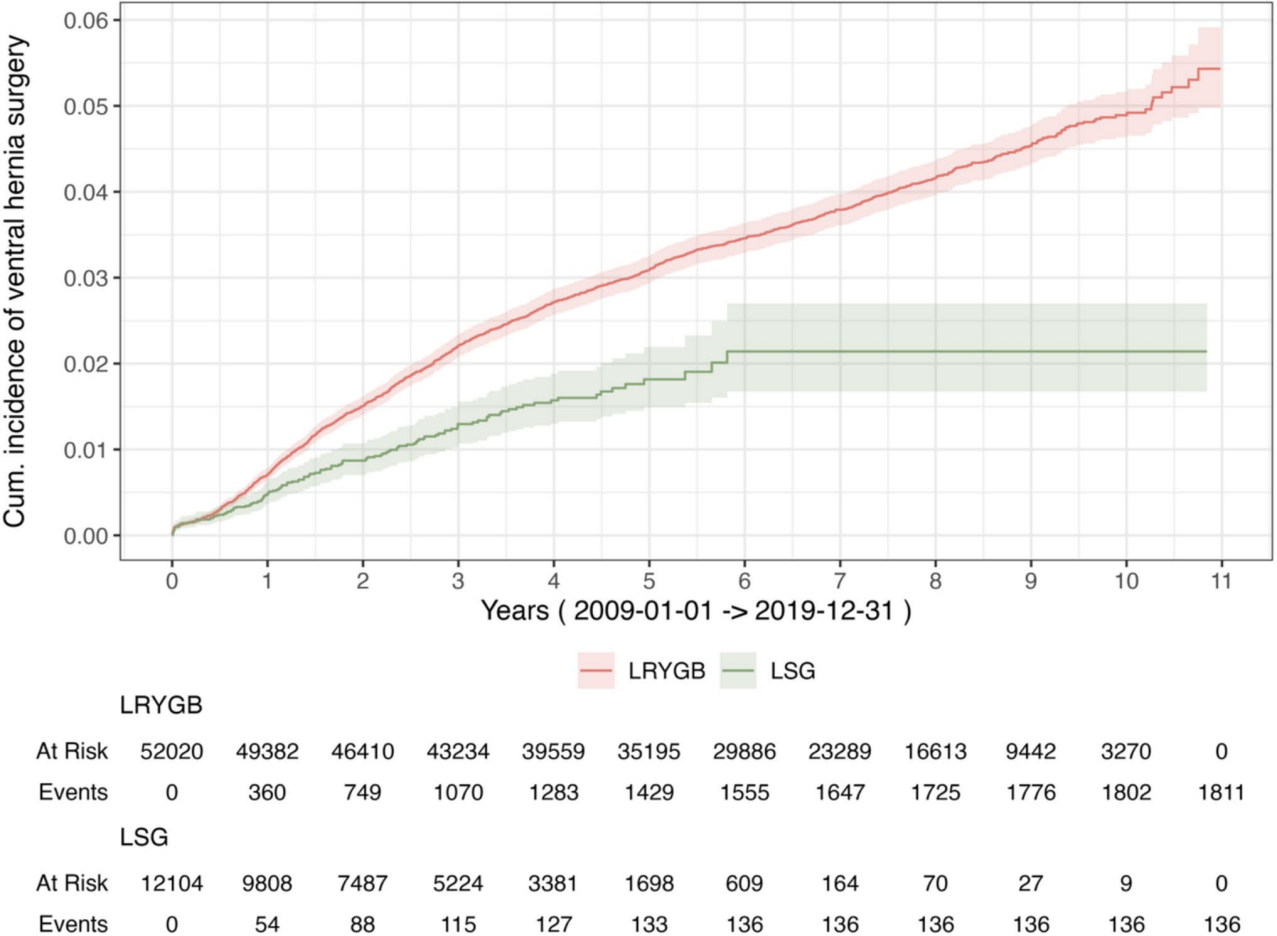
Cumulative incidence of ventral hernia surgery, by methods LRYGB or LSG, 2009–2019. LRYGB; Laparoscopic Roux-en-Y Gastric bypass, LSG; Laparoscopic Sleeve Gastrectomy

**Fig. 5:**
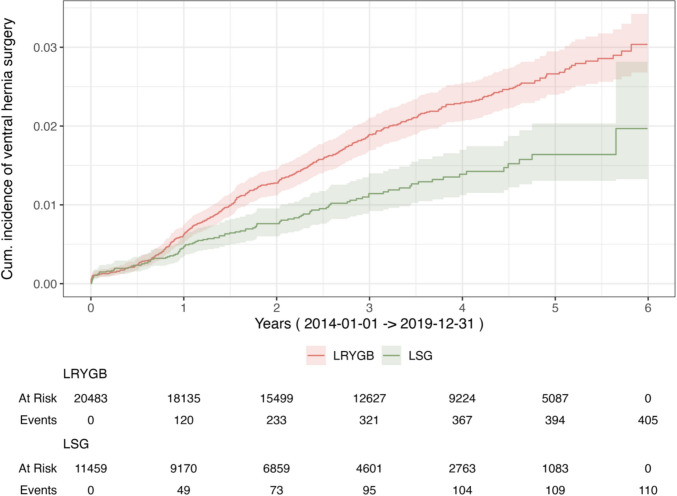
Cumulative incidence of ventral hernia surgery, by methods LRYGB or LSG, 2014–2019. LRYGB; Laparoscopic Roux-en-Y Gastric bypass, LSG; Laparoscopic Sleeve Gastrectomy

The cumulative incidence of ventral hernia surgery at five years was 2.9% (CI 2.8–3.1) (Fig. 3). The overall incidence was 4.7% (CI 4.5–5.0). The probability of having hernia surgery was significantly higher for LRYGB compared to LSG (Breslow test, *p <* 0.001) (Fig. 4). The period 2014–2019 when the proportion of obesity procedures were 64% LRYGB is illustrated in Fig. 5**.**, still showing a statistically significant difference in events between methods (Breslow test, p = 0.001)**.** In the Cox regression model including an interaction between surgical method and time (log-transformed), the interaction term was statistically significant (*p <* 0.001), indicating that the effect of method on risk for ventral hernia surgery varied over time.

Mean time interval between the bariatric procedure and ventral hernia surgery was 36 ± 28 months (range 0–129) in total, for LRYGB 37 ± 28 months (range 0–129) and for LSG 20 ± 16 (range 0–70), *p <* 0.001.

The 5-year cumulative incidence of ventral hernia diagnosis after bariatric surgery was 3.2% (CI 3.1–3.4) in the whole cohort.

Out of the patients with at least one ventral hernia ICD-code 2009–2019, 1740/2906 (60%) had a KVÅ-code for ventral hernia surgery. Of the total cohort 568/64 124 (0.9%) had an ICD-code registered 2009–2019 but prior to their obesity surgery, corresponding to 200/1947 (10%) of the subsequent registered KVÅ-codes. Among the 1947 patients that had at least one ventral hernia surgery after their bariatric procedure, 1513 (77%) had a ventral hernia diagnosis registered after the obesity surgery. On the date of the obesity procedure, 186 patients had a KVÅ-code for ventral hernia, and 136 had a ventral hernia ICD-code, only 80 patients had both. The proportion of concomitant surgery for incisional hernia or umbilical hernia at the obesity surgery registered in SOReg that had a ventral hernia KVÅ-code registered in NPR at the date of obesity surgery was 147/227 (65%).

The most common first registered ventral hernia KVÅ-code in this material, presented by group, was repair of incisional hernia (JAD) with 1010/1947 (52%), followed by repair of umbilical hernia (JAF) with 695/1947 (36%), repair of other hernias and defects of the abdominal wall (JAG) with 235/1947 (12%) and repair of epigastric hernia (JAE) with 152/1947 (8%).

## Discussion

### Incidence

This registry based national study of more than 64 000 patients showed a cumulative five-year incidence of nearly 3% for ventral hernia repair after bariatric surgery.

Our reported incidence at five years is lower than studies on TSH using imaging, as would be expected, but higher than several previous retrospective studies based on rate of hernia repair rather than on objective examination [21, 22]. As the diagnosing of a TSH can be challenging, the true incidence of ventral hernia such as trocar site hernia may be higher than observed in our cohort. As previously stated, the proportion of symptomatic trocar site hernias remains unknown. Although this material included data of hernia diagnosis, the uncertainty in accuracy and completeness of coding limits the ability to draw meaningful conclusions about the proportion of hernias with symptoms. The hernias subjected to surgery – presumably representing those of clinical importance – were the focus of this study.

The inclusion period was selected to allow for long term follow-up, in line with European Hernia Society guidelines for open surgery, though a recommendation for laparoscopy is not yet in place [23].

The European Hernia Society classification of ventral hernias so far distinguishes between primary and incisional hernias [24], but we await a more stringent terminology. We know from other studies that after laparoscopy, the umbilical trocar site is at risk of TSH. Also, the presence of a pre-existing umbilical hernia is a risk factor for a TSH at the umbilicus [25, 26]. The nomenclature is confusing here; in the case of a pre-existing umbilical hernia, is it then a recurrent umbilical hernia or a TSH? For this study, we cannot make such distinguishments. The most common procedure codes in this material were “repair of incisional hernia” followed by “repair of umbilical hernia”. The ambiguity in correct hernia coding brings uncertainty in our material to which kind of hernia was intended, and further conclusions regarding the specific combinations of codes used was not drawn.

### Difference between methods

We found a difference in incidence of surgery for ventral hernia between the two bariatric surgery methods used in Sweden, with a higher rate for LRYGB.

As the use of LSG did not start until 2012, there was a difference in follow-up between groups. Although method remained a significant predictor at baseline, the difference in risk decreased with increasing follow-up. Between 2009 and 2019, potential factors not accounted for in this study might have affected the risk of hernia in need of surgical repair.

A difference between the LRYGB and LSG groups regarding some of the known risk factors [27, 28] was seen in this material (Table 1) but was not further analyzed as this was out of the scope of the present study. Considering technical risk factors for hernia development, a lower rate for LSG is somewhat surprising as this method requires the extraction of a specimen through one of the trocar sites, making it a site at risk of TSH [29]. One possible explanation could be a higher tendency to suture a larger defect. TSH risk factors associated to trocars have previously been studied by several authors [5, 28, 30]. Unfortunately, the registries used in this study do not contain data of trocar site, nor trocar size, type or number of trocars. Furthermore, SOReg lacks data on entry technique and whether the trocar sites were closed or not.

As the hernia surgery incidence was higher for LRYGB in this study, an upcoming study will focus on whether the difference between methods still stands when taking into account other abdominal surgeries and adjusting for general risk factors associated with TSH, such as patient-related (i.e. obesity, smoking or diabetes mellitus) and surgery-related factors (i.e. duration of surgery or complications such as surgical site infection) [5, 28].

Further, we need to relate our findings to the incidence of ventral hernia surgery in a general population [31]. In a recent retrospective study from Australia, the two-decade cumulative population-adjusted incidence of incisional and ventral hernia repair was 182 per 100,000. It varied by hernia type and was highest for umbilical hernias, followed by incisional hernias [32]. The populations should not be directly compared, but it gives a hint of the risk of hernia repair in a high-income country. Worldwide, it is estimated that nearly 580,000 people undergo bariatric surgery annually [16], but along with the increased prevalence of morbid obesity, minimally invasive surgery in any surgical field will encounter patients with obesity-associated surgical and anesthesiologic risk factors. A nationwide French register study by Moszkowicz et al. examining ventral hernia repair and recurrence in bariatric surgery patients reported a 10-year recurrence rate of 23.4% [33]. As in our study, the use of procedural codes did not allow for differentiation between trocar site hernias and other ventral hernias. Neverthless, the observed high rate of reoperations provides valuable insight as it highlights the clinical relevance of ventral hernias among patients with obesity.

Regardless of the true type of ventral hernia – trocar site hernia or other – if nearly 3% of bariatric surgery patients need surgical ventral hernia repair at five years of follow-up, methods to prevent hernia formation should be investigated and developed.

### Limitations

Our results must be interpreted with caution due to limitations related to any observational, nonrandomized study with retrospectively collected data, such as the risk of undetected confounding and bias.

More specific limitations are the lack of precision that inevitably comes with the use of codes other than the ones we aim to study, and the lack of data of other abdominal surgeries or pre-existing ventral hernias. The decision not to include data on other abdominal surgeries from NPR was made to ensure manageable data handling and maintain focus on the primary research question. However, since previous abdominal surgery is a known risk factor for hernia formation, this exclusion limits the ability to fully assess causality. Obesity but also previous bariatric surgery are risk factors for cholelithiasis and risk of subsequent cholecystectomy [32]. Previous cholecystectomy is registered in SOReg, but data of this procedure after the bariatric one is lacking in our material. Another example of post-bariatric surgery not represented in our dataset is surgery for bowel obstruction related to mesenteric defects following gastric bypass [34].

## Limitations related to obtainable variables

To this date, the ICD- and KVÅ classifications lack specific codes for trocar site hernia ortrocar site hernia repair. Proxy procedures were selected to capture trocar site hernia repairs coded as umbilical- incisional- or other hernia repairs. This may have led to an overestimated incidence of TSH, and our findings should be interpreted as reflecting ventral hernia surgeries, among which TSHs are likely present.

A discrepancy was observed between KVÅ- and ICD-codes, precluding firm conclusions about the proportion of hernias that required surgical repair compared with those that did not.

The NPR does not include primary care, and possible hernia diagnoses registered there are consequently not included in this study.

Regarding SOReg, there was a significant amount of incomplete or missing data in the follow-up registrations, making analyses from post-operative data less accurate and hence not presented in the manuscript.

## Conclusion

The cumulative 5-year incidence of surgery for ventral hernia after bariatric surgery was 2.9%. The proportion of true trocar site hernias among them cannot be stated from this study. The ventral hernia incidence differs between the methods gastric bypass and sleeve gastrectomy, with a higher rate for LRYGB.

## Electronic supplementary material

Below is the link to the electronic supplementary material.

Supplementary file1 (PDF 1982 KB)

## Appendix

**Supplementary Table A:** Classification of Health Care Procedures (KVÅ; Classification of Health Care Measures) requested in the data retrieval from the National Patient Register (NPR)

JAD	Repair of incisional hernia
JAD10	Repair of incisional hernia using prosthetic material
JAD11	Laparoscopic repair of incisional hernia using prosthetic material
JAD13	Repair of incisional hernia using prosthetic material and relieving incision*
JAD20	Repair of incisional hernia using prostethic material (onlay)
JAD23	Repair of incisional hernia using prostethic material (onlay) and relieving incision*
JAD30	Repair of incisional hernia using prosthetic material (interstitial)
JAD33	Repair of incisional hernia using prosthetic material (interstitial) and relieving incision*
JAD40	Repair of incisional hernia using prostethic material (sublay)*
JAD41	Laparoscopic repair of incisional hernia using prostethic material (sublay)*
JAD43	Repair of incisional hernia using prostethic material (sublay) and relieving incision*
JAD47	Laparoscopic repair of incisional hernia using prostethic material (sublay) and relieving incision*
JAD50	Repair of incisional hernia using prosthetic material (IPOM)*
JAD51	Laparoscopic repair of incisional hernia using prosthetic material (IPOM)*
JAD60	Repair of incisional hernia using prosthetic material (inlay)*
JAD61	Laparoscopic repair of incisional hernia using prosthetic material (inlay)*
JAD80	Other repair of incisional hernia*
JAD81	Other laparoscopic repair of incisional hernia*
JAD96	Other repair of incisional hernia **
JAD97	Other laparoscopic repair of incisional hernia**
JAE	**Repair of epigastric hernia**
JAE10	Repair of epigastric hernia using suture
JAE11	Laparoscopic repair of epigastric hernia using suture*
JAE20	Repair of epigastric hernia using prosthetic material (onlay)*
JAE30	Repair of epigastric hernia using prosthetic material (interstitial)*
JAE40	Repair of epigastric hernia using prosthetic material (sublay)*
JAE41	Laparoscopic repair of epigastric hernia using prosthetic material (sublay)*
JAE50	Repair of epigastric hernia using prosthetic material (IPOM)*
JAE51	Laparoscopic repair of epigastric hernia using prosthetic material (IPOM)*
JAE60	Repair of epigastric hernia using prosthetic material (inlay)*
JAE61	Laparoscopic repair of epigastric hernia using prosthetic material (inlay)*
JAE71	Laparoscopic repair of epigastric hernia using prosthetic material (multiple layers)*
JAE81	Other laparoscopic repair of epigastric hernia*
JAF	**Repair of umbilical hernia Includes: Paraumbilical hernia**
JAF10	Repair of umbilical hernia using suture
JAF11	Laparoscopic repair of umbilical hernia using suture
JAF20	Repair of umbilical hernia using prosthetic material (onlay)
JAF30	Repair of umbilical hernia using prosthetic material (interstitial)
JAF40	Repair of umbilical hernia using prosthetic material (sublay)*
JAF41	Laparoscopic repair of umbilical hernia using prosthetic material (sublay)*
JAF50	Repair of umbilical hernia using prosthetic material (IPOM)*
JAF51	Laparoscopic repair of umbilical hernia using prosthetic material (IPOM)*
JAF60	Repair of umbilical hernia using prosthetic material (inlay)*
JAF61	Laparoscopic repair of umbilical hernia using prosthetic material (inlay)*
JAF70	Repair of umbilical hernia using prosthetic material (multiple layers)*
JAF80	Other repair of umbilical hernia*
JAF81	Other laparoscopic repair of umbilical hernia*
JAF96	Other repair of umbilical hernia**
JAF97	Other laparoscopic repair of umbilical hernia**
JAG	**Repair of other hernias and defects of abdominal wall**
JAG10	Repair of other hernias and defects of abdominal wall using suture
JAG11	Laparoscopic repair of other hernias and defects of abdominal wall using suture*
JAG20	Repair of other hernias and defects of abdominal wall using prosthetic material (onlay)
JAG30	Repair of other hernias and defects of abdominal wall using flap*
JAG40	Repair of other hernias and defects of abdominal wall using prosthetic material (sublay)*
JAG41	Laparoscopic repair of other hernias and defects of abdominal wall using prosthetic material (sublay)*
JAG50	Repair of other hernias and defects of abdominal wall using prosthetic material (IPOM)*
JAG51	Laparoscopic repair of other hernias and defects of abdominal wall using prosthetic material (IPOM)*
JAG60	Repair of other hernias and defects of abdominal wall using prosthetic material (inlay)
JAG61	Laparoscopic repair of other hernias and defects of abdominal wall using prosthetic material (inlay)*
JAG80	Other repair of other hernias and defects of abdominal wall*
JAG81	Other laparoscopic repair of other hernias and defects of abdominal wall*
JAG96	Other reconstruction of abdominal wall**
JAG97	Other laparoscopic reconstruction of abdominal wall**

*new code since 2012–01-01; ** code expired 2012–01-01

**Supplementary Table B:** Discharge diagnoses according to the current Swedish version of The International Classification of Diseases, Tenth Revision (ICD-10-SE) requested in the data retrieval from the National Patient Register (NPR)

K42	Umbilical hernia
K42.0	Umbilical hernia with obstruction, without gangrene
K42.1	Umbilical hernia with gangrene
K42.9	Umbilical hernia without obstruction or gangrene
K43	Ventral hernia
K43.0	Incisional hernia with obstruction, without gangrene
K43.1	Incisional hernia with gangrene
K43.2	Incisional hernia without obstruction or gangrene
K43.6	Other and unspecified ventral hernia with obstruction, without gangrene
K43.7	Other and unspecified ventral hernia with gangrene
K43.9	Ventral hernia without obstruction or gangrene
