# Supplementary material for: Incidence of ventral hernia surgery after laparoscopic bariatric surgery in Sweden: a registry-based study 2009–2019
Source: Hernia. 2025 Dec 20;30(1):43. doi: 10.1007/s10029-025-03547-w (PMC12718248; doi:10.1007/s10029-025-03547-w)
Supplement: Supplementary file 1 — Supplementary file1 (PDF 1982 KB) [file 10029_2025_3547_MOESM1_ESM.pdf]

Metadata of the article that will be visualized online

ArticleTitle		Incidence of ventral hernia surgery after laparoscopic bariatric surgery in sweden: a registry-based study 2009–2019
Article CopyRight - Year		The Author(s), under exclusive licence to Springer-Verlag France SAS, part of Springer Nature 2025

Corresponding Author	Family Name	Ahlqvist
	Particle	
	Given Name	Correspondance Sandra
	Organization	Umeå University, Region Västernorrland
	Address	Sundsvall 851 86, Sweden, SWEDEN
	Division	Dept of Diagnostics and Intervention, Sundsvall Hospital, Länsorganisationen för Operation och Intensivvård
	Email	sandra.ahlqvist@umu.se
Author	Family Name	AHLQVIST
	Particle	
	Given Name	Sandra
	Organization	Umeå University
	Address	Sweden
	Division	Department of Diagnostics and Intervention (Sundsvall Hospital)
	Email	
Author	Family Name	WALLDÉN
	Particle	
	Given Name	Jakob
	Organization	Umeå University
	Address	Sweden
	Division	Department of Diagnostics and Intervention (Sundsvall Hospital)
	Email	
Author	Family Name	DACKHAMMAR
	Particle	
	Given Name	Johan BLIXT
	Organization	Danderyd Hospital, Karolinska Institutet
	Address	Stockholm, Sweden
	Division	Department of Clinical Sciences
	Email	
Author	Family Name	DACKHAMMAR
	Particle	

	Given Name	Johan BLIXT
	Organization	Sundsvall Hospital
	Address	Sweden
	Email	
Author	Family Name	NORDIN
	Particle	
	Given Name	Pär
	Organization	Umeå University
	Address	Sweden
	Division	Department of Diagnostics and Intervention (Östersund Hospital)
	Email	
Author	Family Name	WADSTEN
	Particle	
	Given Name	Charlotta
	Organization	Umeå University
	Address	Sweden
	Division	Department of Diagnostics and Intervention (Sundsvall Hospital)
	Email	
Author	Family Name	Ottosson
	Particle	
	Given Name	Johan
	Organization	Örebro University
	Address	Örebro, Sweden
	Division	Department of Surgery, Faculty of Medicine and Health
	Email	
Author	Family Name	CENGIZ
	Particle	
	Given Name	Yücel
	Organization	Umeå University
	Address	Sweden
	Division	Department of Diagnostics and Intervention (Sundsvall Hospital)
	Email	
Schedule	Received	30 September 2025
	Revised	
	Accepted	21 November 2025

Hernia _####################_
https://doi.org/10.1007/s10029-025-03547-w

ORIGINAL ARTICLE

Incidence of ventral hernia surgery after laparoscopic bariatric surgery in sweden: a registry-based study 2009–2019

Sandra AHLQVIST[1] · Jakob WALLDÉN[1] · Johan BLIXT DACKHAMMAR[2,3] · Pär NORDIN[4] · Charlotta WADSTEN[1] · Johan Ottosson[5] · Yücel CENGIZ[1] · Correspondance Sandra Ahlqvist[6]

Received: 30 September 2025 / Accepted: 21 November 2025
© The Author(s), under exclusive licence to Springer-Verlag France SAS, part of Springer Nature 2025

UNCORRECTED PROOF

✉ Correspondance Sandra Ahlqvist
sandra.ahlqvist@umu.se

1 Department of Diagnostics and Intervention (Sundsvall Hospital), Umeå University, Sweden

2 Department of Clinical Sciences, Danderyd Hospital, Karolinska Institutet, Stockholm, Sweden

3 Sundsvall Hospital, Sweden

4 Department of Diagnostics and Intervention (Östersund Hospital), Umeå University, Sweden

5 Department of Surgery, Faculty of Medicine and Health, Örebro University, Örebro, Sweden

6 Dept of Diagnostics and Intervention, Sundsvall Hospital, Länsorganisationen för Operation och Intensivvård, Umeå University, Region Västernorrland, Sundsvall 851 86, Sweden, SWEDEN

AQ3

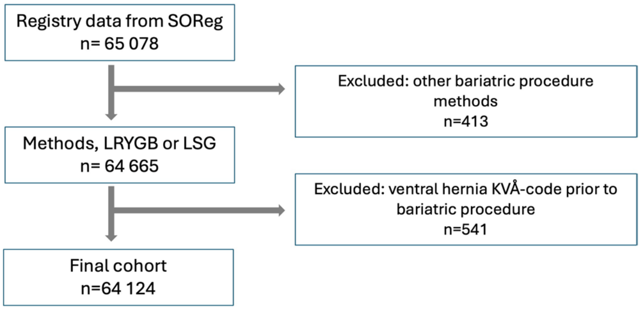
Registry data from SOReg
n= 65 078
Excluded: other bariatric procedure methods
n=413
Methods, LRYGB or LSG
n= 64 665
Excluded: ventral hernia KVÅ-code prior to bariatric procedure
n=541
Final cohort
n=64 124

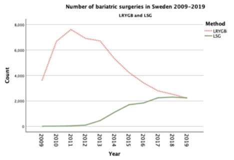
Number of bariatric surgeries in Sweden 2009–2019
LRYGB and LSG
Method
Count
Year

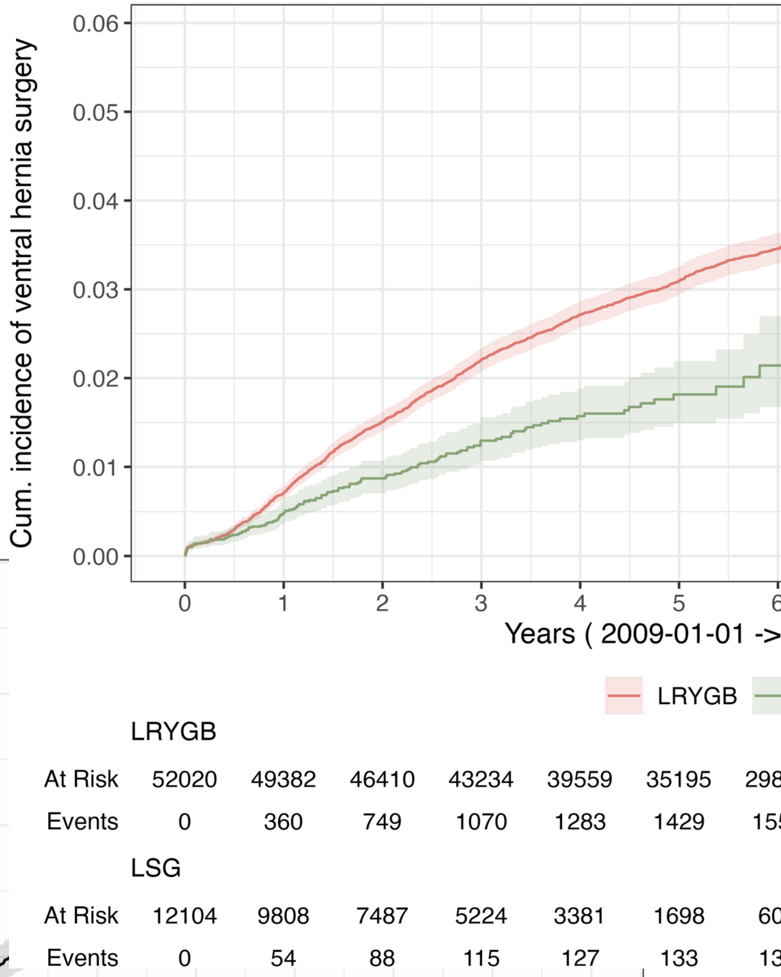
Cum. incidence of ventral hernia surgery
0.06
0.05
0.04
0.03
0.02
0.01
0.00
0
1
2
3
4
5
Years (2009-01-01 ->
LRYGB
LRYGB
At Risk 52020 49382 46410 43234 39559 35195
Events 0 360 749 1070 1283 1429
LSG
At Risk 12104 9808 7487 5224 3381 1698
Events 0 54 88 115 127 133

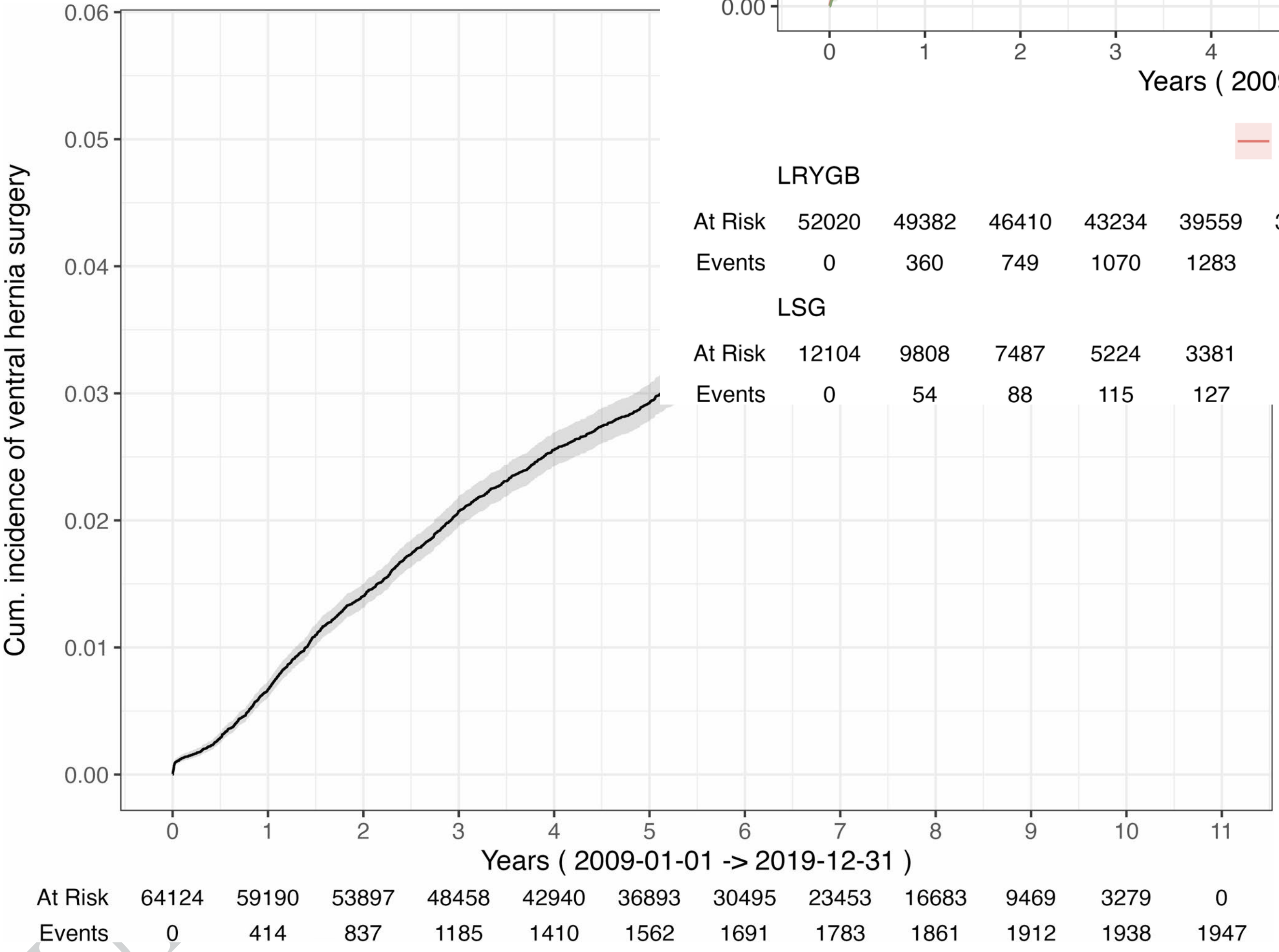
Cum. incidence of ventral hernia surgery
0.06
0.05
0.04
0.03
0.02
0.01
0.00
0 1 2 3 4 5 6 7 8 9 10 11
Years (2009-01-01 -> 2019-12-31)
At Risk 64124 59190 53897 48458 42940 36893 30495 23453 16683 9469 3279 0
Events 0 414 837 1185 1410 1562 1691 1783 1861 1912 1938 1947

Table 1 Patient characteristics

	Total	LRYGB	LSG	
	n = 64 124	*n* = 52 020 (81.1%)	*n* = 12 104 (18.9%)	
BMI at bariatric surgery (mean, SD)	41.6 (5.5)	42.1 (5.3)	39.6 (5.8)	$p < 0.001$
Sex, n (%)				
men, n (%)	14 875/64 124 (23.2)	12 548/52 020 (24.1)	2327/12 104 (19.2)	$p < 0.001$
women, n (%)	49 249/64 124 (76.8)	39 472/52 020 (75.9)	9777/12 104 (80.8)	$p < 0.001$
Age at bariatric surgery, years (mean, SD)	41 (11.0)	41 (11.0)	41 (11.0)	$p = 0.096$
OSA, n (%)	6237/64 123 (9.6)	5356/52 019 (10.3)	881/12 104 (7.3)	$p < 0.001$
Arterial hypertension, n (%)	15 797/64 123 (24.6)	13 372/52 019 (25.7)	2425/12 104 (20.0)	$p < 0.001$
Diabetes mellitus, n (%)	8426/64 123 (13.1)	7350/52 019 (14.1)	1076/12 104 (8.9)	$p < 0.001$
Actively smoking, n (%)	6212/53 109 (11.7)	5216/42 005 (12.4)	993/11 104 (9.0)	$p < 0.001$

LRYGB; Laparoscopic Roux-en-Y Gastric bypass, LSG; Laparoscopic Sleeve Gastrectomy, OSA; Obstructive sleep apnea.

*new code since 2012-01-01; ** code expired 2012-01-01.

Table 3 Discharge diagnoses according to the current Swedish version of The International Classification of Diseases, Tenth Revision (ICD-10-SE) requested in the data retrieval from the National Patient Register (NPR).

K42	Umbilical hernia
K42.0	Umbilical hernia with obstruction, without gangrene
K42.1	Umbilical hernia with gangrene
K42.9	Umbilical hernia without obstruction or gangrene
K43	Ventral hernia
K43.0	Incisional hernia with obstruction, without gangrene
K43.1	Incisional hernia with gangrene
K43.2	Incisional hernia without obstruction or gangrene
K43.6	Other and unspecified ventral hernia with obstruction, without gangrene
K43.7	Other and unspecified ventral hernia with gangrene
K43.9	Ventral hernia without obstruction or gangrene

UNCORRECTED PROOF

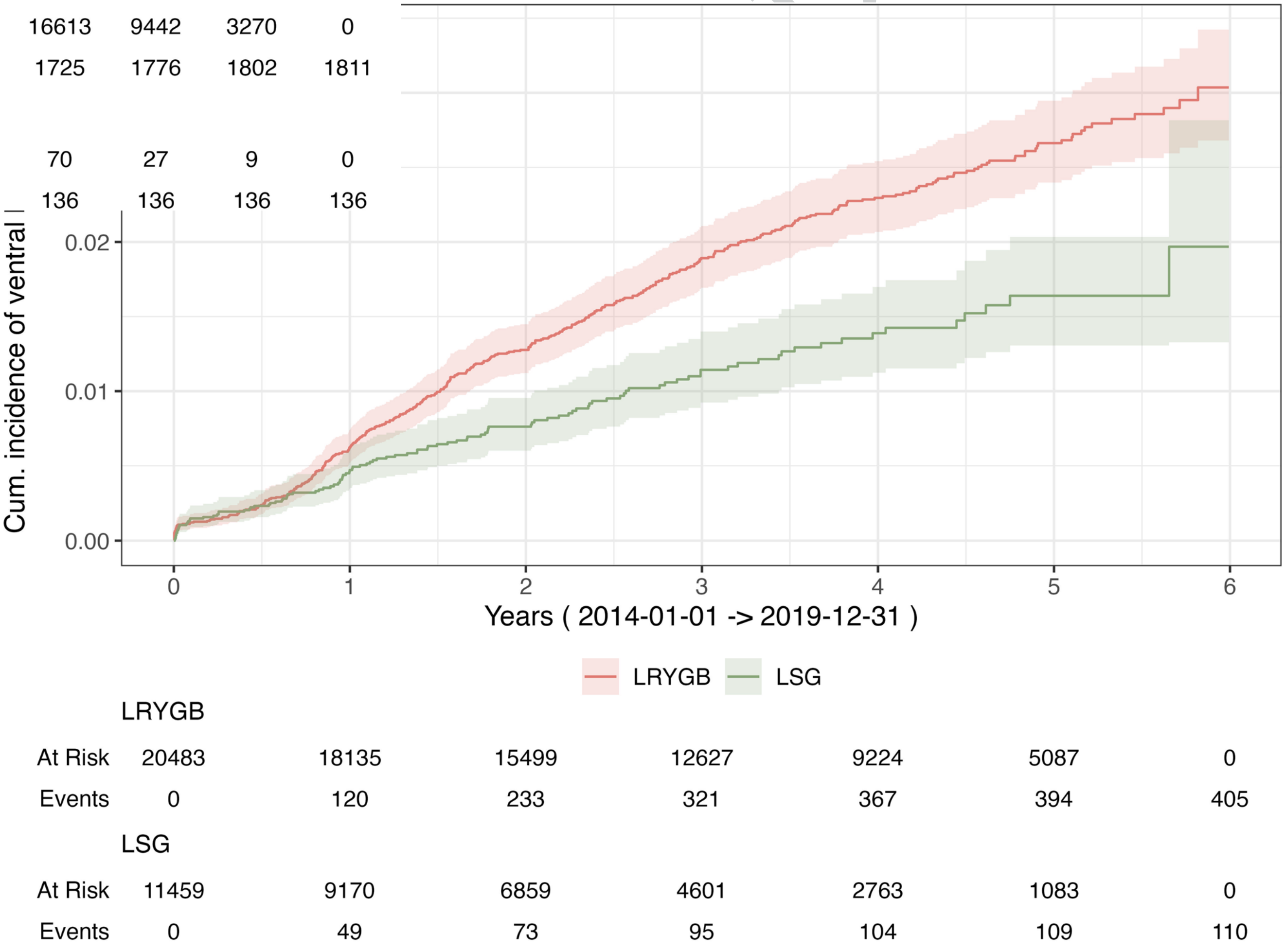

Table 2 Classification of health care procedures (KVÅ; classification of health care Measures) requested in the data retrieval from the National patient register (NPR)

JAD	Repair of incisional hernia
JAD10	Repair of incisional hernia using prosthetic material
JAD11	Laparoscopic repair of incisional hernia using prosthetic material
JAD13	Repair of incisional hernia using prosthetic material and relieving incision*
JAD20	Repair of incisional hernia using prostethic material (onlay)
JAD23	Repair of incisional hernia using prostethic material (onlay) and relieving incision*
JAD30	Repair of incisional hernia using prosthetic material (interstitial)
JAD33	Repair of incisional hernia using prosthetic material (interstitial) and relieving incision*
JAD40	Repair of incisional hernia using prostethic material (sublay)*
JAD41	Laparoscopic repair of incisional hernia using prostethic material (sublay)*
JAD43	Repair of incisional hernia using prostethic material (sublay) and relieving incision*
JAD47	Laparoscopic repair of incisional hernia using prostethic material (sublay) and relieving incision*
JAD50	Repair of incisional hernia using prosthetic material (IPOM)*
JAD51	Laparoscopic repair of incisional hernia using prosthetic material (IPOM)*
JAD60	Repair of incisional hernia using prosthetic material (inlay)*
JAD61	Laparoscopic repair of incisional hernia using prosthetic material (inlay)*
JAD80	Other repair of incisional hernia*
JAD81	Other laparoscopic repair of incisional hernia*
JAD96	Other repair of incisional hernia **
JAD97	Other laparoscopic repair of incisional hernia**
JAE	**Repair of epigastric hernia**
JAE10	Repair of epigastric hernia using suture
JAE11	Laparoscopic repair of epigastric hernia using suture*
JAE20	Repair of epigastric hernia using prosthetic material (onlay)*
JAE30	Repair of epigastric hernia using prosthetic material (interstitial)*
JAE40	Repair of epigastric hernia using prosthetic material (sublay)*
JAE41	Laparoscopic repair of epigastric hernia using prosthetic material (sublay)*
JAE50	Repair of epigastric hernia using prosthetic material (IPOM)*
JAE51	Laparoscopic repair of epigastric hernia using prosthetic material (IPOM)*
JAE60	Repair of epigastric hernia using prosthetic material (inlay)*
JAE61	Laparoscopic repair of epigastric hernia using prosthetic material (inlay)*
JAE71	Laparoscopic repair of epigastric hernia using prosthetic material (multiple layers)*
JAE81	Other laparoscopic repair of epigastric hernia*
JAF	**Repair of umbilical hernia Includes: Paraumbilical hernia**
JAF10	Repair of umbilical hernia using suture
JAF11	Laparoscopic repair of umbilical hernia using suture
JAF20	Repair of umbilical hernia using prosthetic material (onlay)
JAF30	Repair of umbilical hernia using prosthetic material (interstitial)
JAF40	Repair of umbilical hernia using prosthetic material (sublay)*
JAF41	Laparoscopic repair of umbilical hernia using prosthetic material (sublay)*
JAF50	Repair of umbilical hernia using prosthetic material (IPOM)*
JAF51	Laparoscopic repair of umbilical hernia using prosthetic material (IPOM)*
JAF60	Repair of umbilical hernia using prosthetic material (inlay)*
JAF61	Laparoscopic repair of umbilical hernia using prosthetic material (inlay)*
JAF70	Repair of umbilical hernia using prosthetic material (multiple layers)*
JAF80	Other repair of umbilical hernia*
JAF81	Other laparoscopic repair of umbilical hernia*
JAF96	Other repair of umbilical hernia**
JAF97	Other laparoscopic repair of umbilical hernia**
JAG	**Repair of other hernias and defects of abdominal wall**
JAG10	Repair of other hernias and defects of abdominal wall using suture
JAG11	Laparoscopic repair of other hernias and defects of abdominal wall using suture*
JAG20	Repair of other hernias and defects of abdominal wall using prosthetic material (onlay)
JAG30	Repair of other hernias and defects of abdominal wall using flap*
JAG40	Repair of other hernias and defects of abdominal wall using prosthetic material (sublay)*

UNCORRECTED PROOF

Table 2 (continued)

JAD	Repair of incisional hernia
JAG41	Laparoscopic repair of other hernias and defects of abdominal wall using prosthetic material (sublay)*
JAG50	Repair of other hernias and defects of abdominal wall using prosthetic material (IPOM)*
JAG51	Laparoscopic repair of other hernias and defects of abdominal wall using prosthetic material (IPOM)*
JAG60	Repair of other hernias and defects of abdominal wall using prosthetic material (inlay)
JAG61	Laparoscopic repair of other hernias and defects of abdominal wall using prosthetic material (inlay)*
JAG80	Other repair of other hernias and defects of abdominal wall*
JAG81	Other laparoscopic repair of other hernias and defects of abdominal wall*
JAG96	Other reconstruction of abdominal wall**
JAG97	Other laparoscopic reconstruction of abdominal wall**

Supplementary Information The online version contains supplementary material available at https://doi.org/10.1007/s10029-025-03547-w.

Publisher's note Springer Nature remains neutral with regard to jurisdictional claims in published maps and institutional affiliations.

Springer Nature or its licensor (e.g. a society or other partner) holds exclusive rights to this article under a publishing agreement with the author(s) or other rightsholder(s); author self-archiving of the accepted manuscript version of this article is solely governed by the terms of such publishing agreement and applicable law. AQ7

UNCORRECTED PROOF

Journal: **10029**
Article: **3547**

AQ1. City information missing in affiliation for author(s) (Aff1, Aff3, Aff4) in the manuscript. Please check.

AQ2. Please provide missing abstract as per journal submission guidelines.

AQ3. Please provide 5-10 missing keywords as these are required for the journal.

AQ4. Table 1 is not cited in the manuscript. Please insert the table citation at the correct location in the text.

AQ5. Table 2 is not cited in the manuscript. Please insert the table citation at the correct location in the text.

AQ6. Table 3 is not cited in the manuscript. Please insert the table citation at the correct location in the text.

AQ7. Please provide competing interests as per journal submission guidelines.
